# An original study assessing biomarker success rate in breast cancer recurrence biomarker research

**DOI:** 10.1186/s12916-024-03460-6

**Published:** 2024-07-29

**Authors:** K-V. Savva, A. MacKenzie, R. C. Coombes, N. M. Zhifang, B. G. Hanna, C. J. Peters

**Affiliations:** 1 Department of Surgery and Cancer, Imperial College London, 10th Floor QEQM Wing, St Mary’s Hospital, London, W2 1NY UK; 2 Division of Surgery, Department of Surgery and Cancer, Imperial College London, London, UK

**Keywords:** Breast cancer, Biomarkers, Translational research, Clinical utility

## Abstract

**Background:**

Breast cancer is the second most common cause of cancer mortality worldwide. Biomarker discovery has led to advances in understanding molecular phenotyping and thus has a great potential for precision management of this diverse disease. Despite increased interest in the biomarker field, only a small number of breast cancer biomarkers are known to be clinically useful. Therefore, it is very important to characterise the success rate of biomarkers in this field and study potential reasons for the deficit. We therefore aim to achieve quantitative characterisation of the biomarker translation gap by tracking the progress of prognostic biomarkers associated with breast cancer recurrence.

**Methods:**

An electronic systematic search was conducted in Medline and Embase databases using keywords and mesh headings associated with breast cancer recurrence biomarkers (1940–2023). Abstracts were screened, and primary clinical studies involving breast cancer recurrence biomarkers were selected. Upon identification of relevant literature, we extracted the biomarker name, date of publication and journal name. All analyses were performed using IBM SPSS Statistics and GraphPad prism (La Jolla, California, USA).

**Results:**

A total of 19,195 articles were identified, from which 4597 articles reported breast cancer biomarkers associated with recurrence. Upon data extraction, 2437 individual biomarkers were identified. Out of these, 23 are currently recommended for clinical use, which corresponds to only 0.94% of all discovered biomarkers.

**Conclusions:**

This study characterised for the first time the translational gap in the field of recurrence-related breast cancer biomarkers, indicating that only 0.94% of identified biomarkers were recommended for clinical use. This denotes an evident barrier in the biomarker research field and emphasises the need for a clearer route from biomarker discovery through to implementation.

**Supplementary Information:**

The online version contains supplementary material available at 10.1186/s12916-024-03460-6.

## Background

According to GLOBOCAN 2018, breast cancer is the second leading cause of cancer death worldwide [1]. Breast cancer is highly heterogenous and encompasses many distinct biological entities with specific biological features and pathological behaviours [2]. Approximately 30% of patients experience recurrence while 90% of cancer-related deaths are due to metastatic recurrence [3–5]. A range of prognostic factors have been associated with the incidence of recurrence including age, tumour size, stage and lymph node involvement [6].

Discovery of biomarkers associated with cancer is an immensely researched field because of the associated clinical utility they offer; yet discerning which biomarkers are clinically useful is challenging. Indeed, despite the high rate of biomarker discovery, only a small percentage of biomarkers are directly impacting patient care, indicating a shortfall in the route toward translation. This has driven us in developing the Biomarker Toolkit, a tool used to mediate the successful translation of biomarkers from bench to bedside [7].

A more promising biomarker would usually attract academic interest [8]. This would result in an increased number of publications that are of higher quality [8]. Although factors associated with the biomarker translational gap have been discussed by numerous studies, a quantitative assessment of this gap is currently lacking. To this end, this original study primarily aims to quantitatively characterise the scale of biomarker translation gap, in the field of recurrence-related breast cancer biomarkers. This will be achieved by tracking the up-to-date progress of all prognostic biomarkers directly/indirectly associated with breast cancer recurrence. Aiming to examine specific factors associated with biomarker success, we also sought to investigate the relationship between biomarker clinical utilisation and publication quality/quantity (i.e. publication frequency and article impact factor), while suggesting potential solutions to improve biomarker translation rate.

## Methods

### Systematic search

**Fig. 1:**
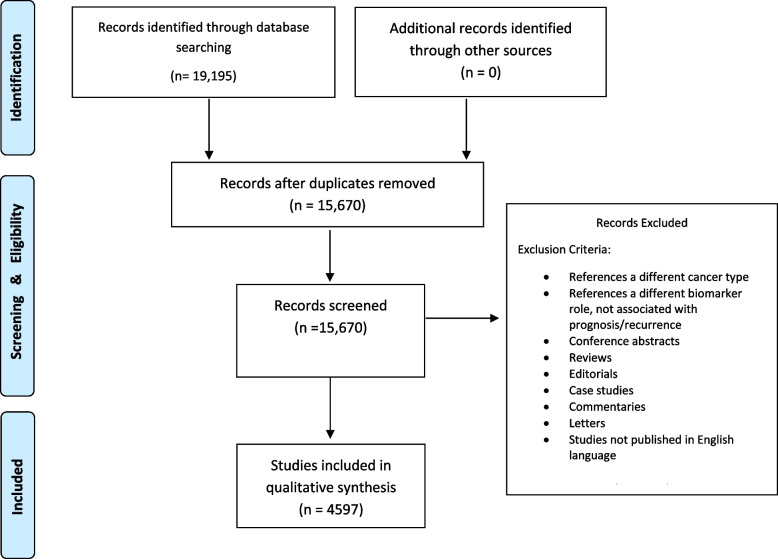
PRISMA illustrating the flow of literature selection for the systematic search

A systematic literature search with no date limit was conducted in Ovid, using Medline and Embase databases, to obtain primary literature linking breast cancer biomarkers and prognosis/recurrence (search conducted in June 2023). Primary clinical studies refer to original research papers which conduct biomarker evaluation using clinical studies. The main keywords used included: “breast cancer”, “biomarker” and “recurrence”. The terms were expanded and adjusted to each database. Broad keywords were selected/utilised in the search in order to avoid losing relevant publications. The full search strategy including all keywords and terms used can be found in Additional file: Table S1. Inclusion criteria included primary studies which considered: breast cancer, a molecular biomarker, reference to recurrence, disease-free survival and/or metastasis. Exclusion criteria included conference abstracts, reviews, editorials, case studies, commentaries, letters and studies not published in the English language. A subset of the biomarkers included in this review are well known to have additional roles. For example, ER and PR are routinely utilised to subtype breast cancer patients. In this review, if any biomarker was associated with breast cancer recurrence-related outcomes, as detailed in the Additional file: Table S1, it was selected for inclusion. Duplicate citations were removed using the referencing software Mendeley. The PRISMA guidelines and flow chart were used to guide the literature search (PRISMA checklist and Fig. 1). Once eligible articles were identified, the date of publication, journal and biomarker name were extracted from the abstracts and were tabulated using Microsoft Excel. Biomarker candidates sharing identical characteristics but referenced by diverse nomenclatures were amalgamated into unified entities. Distinct variations or versions of a biomarker candidate, including assessments using different subtypes, were treated as discrete entities. Furthermore, panels comprising biomarker candidates were regarded as distinct entities, irrespective of potential duplication of previously identified biomarkers within them. Publication screening was conducted by two individual reviewers and any disagreements between reviewers were discussed and resolved (KVS and AM).

### Biomarker publication frequency and survival analysis

All relevant articles were manually grouped by biomarker using Microsoft Excel. The total number of biomarkers identified and the total publication frequency for each biomarker were recorded. A frequency bar chart was constructed to identify the publication frequencies for all biomarkers. Biomarkers were grouped into 1, 2–5, 6–10, 11–20 and > 20 publications and the frequency of biomarkers in each category was plotted. Time difference (years) was calculated between the first publication and each subsequent publication for each biomarker, to allow a fair comparison between biomarkers published in different years.

### Clinical use data

All identified biomarkers were separated into two groups: (i) successful and (ii) stalled biomarkers. Successful biomarkers were defined as biomarkers which have been recommended/approved by any of the following guidelines/regulatory bodies: NICE guidelines [9–11], ASCO Clinical Practice Guidelines [12], FDA [13], NCCN [14], European Group on Tumour Markers (EGTM) [15] and St Gallen Consensus [16] (Table 1). Conversely, stalled biomarkers are defined as biomarkers which are (i) not recommended/approved by the previously mentioned guidelines/regulatory authorities and (ii) are not clinically utilised.

**Table 1:** Table indicating successful biomarkers as recommended by EGTM, ASCO, St. Gallen’s consensus and NCCN

	EGTM	ASCO	St.Gallen	NICE	FDA	NCCN
ER	X	X	X	X	X	X
PR	X	X	X	X	X	X
Ki-67	X		X			
HER2	X	X	X	X	X	X
OncoDX	X	X	X	X		X
MammaPrint	X		X		X	X
BCI	X	X	X			X
Prosigna	X	X	X	X	X	X
Endopredict	X	X	X	X		X
UPA and PAI-1	X	X				
CEA	X	X			X	X
CA15-3	X	X			X	X
CA 27–29		X			X	X
Luminal A			X			
Luminal B			X			
TNBC			X			
Basal			X			
BRCA1/2		X	X	X	X	
PIK3CA			X		X	
TILs			X			
P53		X		X		
CTC					X	
PDL1					X	

*ER* estrogen receptors, *PR* progesterone receptors, *HER2* human epidermal growth factor receptor 2, *OncoDx* OncotypeDx, *BCI* Breast Cancer Index, *UPA* urokinase plasminogen activator, *PAI-1* plasminogen activator inhibitor type-1, *CEA* carcinoembryonic antigen, *CA15-3* cancer antigen 15–3, *CA 27–29* cancer antigen, *TNBC* triple negative breast cancer, *BRCA1/2* BReast CAncer gene 1/2, *PIK3CA* phosphatidylinositol-4,5-bisphosphate 3-kinase catalytic subunit alpha, *TILs* tumour-infiltrating lymphocytes, *P53* tumour protein p53, *CTC* circulating tumour cells, *PDL1* programmed death-ligand 1

### Identifying any relation between journal impact factor and biomarker clinical implementation

Journal impact factors for all publications were retrieved from an online database [17] (https://www.scijournal.org/). The highest impact factor publication was selected for each biomarker. A box plot was constructed to illustrate the maximum, median and minimum highest journal impact factor, for both successful and stalled biomarker groups.

### Evaluating differences between stalled and successful biomarker prognostic outcomes using cBioPortal

In an attempt to compare the survival outcomes related with specific biomarker-associated mutations, cBioPortal was utilised. Survival outcomes of all successful and stalled biomarkers associated with mutations with more than 12 publications were assessed in cBioPortal (https://www.cbioportal.org/). We have considered all identified biomarkers with > 12 publications, irrespective of biomarker nature. *P*-values comparing the survival outcome between the mutant and wildtype version of the biomarker from 16 breast cancer studies (6805 breast samples/6391 patients) were extracted, details are listed in Additional File: Table S3. *P*-values were subsequently compared between the stalled and successful biomarkers.

### Statistical analysis

All data analysis was conducted using Graphpad Prism (La Jolla, CA, USA) and IBM SPSS Statistics version 26.0 (IBM Corp., Armonk, NY, USA). *P* value ≤ 0.05 was used to denote significance. IBM SPSS Statistics was used to run a Kolmogorov–Smirnov test and assess data normality. A Mann–Whitney *U* test was applied to evaluate differences between the two groups for both average publication frequency and journal impact factor. A random selection function was employed to procure ten distinct sets of 23 values each for (a) publication frequency and (b) impact factor from the stalled biomarker group. Random function was conducted to select ten batches of stalled biomarkers, aligning their count with that of the successful group, thereby facilitating statistical analysis. Binary logistic regression was conducted to assess the possible correlation between biomarker success and (i) publication frequency, (ii) cBioPortal prognostic data, and (iii) publication impact factor. All graphs were produced in GraphPad Prism 7 (La Jolla, CA, USA).

## Results

Upon identification and screening of 19,195 articles, 4597 studies were selected as they studied breast cancer biomarkers which (i) provided additional prognostic information in terms of recurrence as compared to conventional biomarkers or (ii) could be used as predictive for benefit from a particular therapy associated with recurrence. A detailed process of literature selection is illustrated in the PRISMA flow diagram shown in Fig. 1. Upon data extraction, 2437 individual biomarkers were identified.

**Fig. 2:**
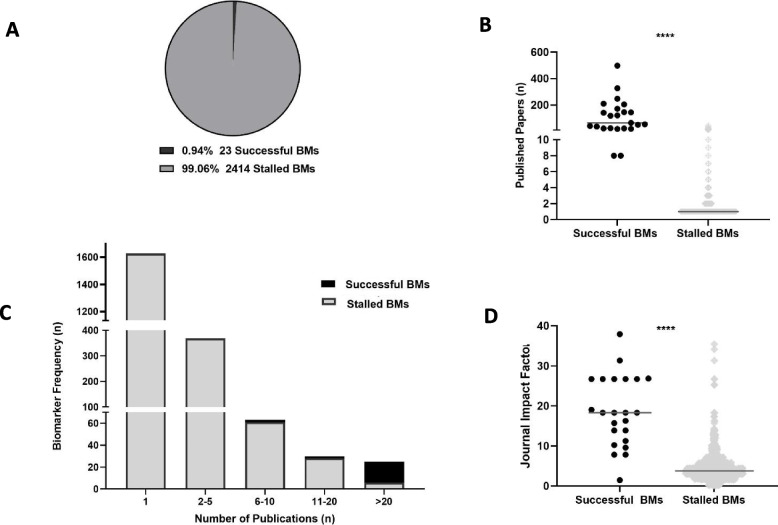
Success rate of breast cancer recurrence biomarkers. **A** Pie chart indicating % of clinically adopted breast cancer recurrence biomarkers. **B** Box plot indicating the median number of published papers, taking into consideration all data points for successful (*n* = 23) and stalled (*n* = 2091) biomarkers **C** Biomarkes frequency bar chart illustrating the number of biomarkers for publication ranges 1, 2–5, 6–10, 11–20 and > 20 (*n* = 2115). **D** Box plot indicating the median highest impact factos. Taking into consideration all data points for each successful and stalled biomarkers. The upper and lower limits of the plots represent max and min values. Successful biomarker (i) publication frequency and (ii) impact factor cohorts (*n* = 23) were compared to ten independent randomly selected stalled biomarkers. In every separate comparison for both publication frequency and impact factor, using each subset of randomly selected data, the Mann–Whitney *U* test yielded a *P*-value of < 0.0001. Asterisks shown in the graph indicate the level of significance where ns: *P* > 0.05 *: *P* ≤ 0.05, **: *P* ≤ 0.01, ***: *P* ≤ 0.001, **** *P* ≤ 0.0001

Out of the 2437 biomarkers identified, 23 are currently recommended for clinical use, which accounts for only 0.94% of all discovered breast cancer recurrence biomarkers (Fig. 2A). Moreover, differences in impact factor and publication frequency were investigated between stalled and successful biomarkers. Average published literature was significantly higher in successful biomarkers with a median of 79 published papers for successful and 1 publication for the stalled biomarker group (Fig. 2B). As indicated in Fig. 2C, there is an extremely high frequency of biomarkers with a single publication in the stalled biomarker group (77.34%). In contrast, the successful biomarker group did not have any biomarkers with a single publication, while 91.7% biomarkers had > 20 publications. The average highest impact factor of successful biomarkers, as indicated by Fig. 2D, was significantly higher in comparison to the stalled biomarker group (*p* ≤ 0.0001). Interestingly, the maximum journal impact factor recorded for the successful biomarkers was 51.77, a biomarker published in the *Annals of Oncology* in 2021 (Ki-67) [18]. Although Fig. 2B and D display all individual data points for the respective graphs, the statistical analysis employed a random selection function to obtain ten distinct sets of 23 values each for (a) publication frequency and (b) impact factor from the stalled biomarker group. The random selection process was conducted to choose ten batches of stalled biomarkers, ensuring their count matched that of the successful group, thereby facilitating statistical analysis. In support, publication frequency and journal impact factor were significantly associated with clinically successful biomarkers, using binary logistic regression (publication frequency: *p* = 0.002 (95% CI: (0.904–0.978)), impact factor: *p* ≤ 0.0001, 95% CI: (0.778–0.890)).

**Fig. 3:**
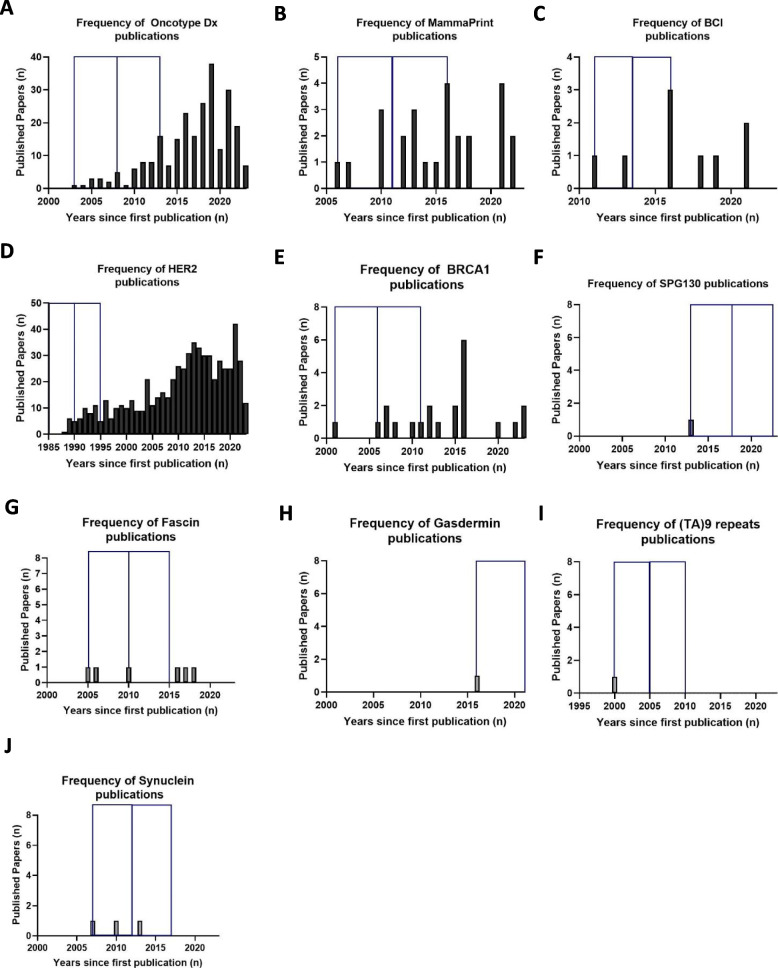
Biomarker Survival Analysis. Bar charts indicating the number of published papers over the years for **A**–**E** Successful and **F**–**J** stalled biomarkers. Random function of Excel was used to select five biomarkers for each group. Blue box squares represent 5- and 10-year windows since first publication (first square = 0–5-year window, second square = 5–10-year window). BCI, Breast Cancer Index, HER2, human epidermal growth factor receptor 2; SPG130, solouble glucoprotein

To compare the publication timeline between the two groups, a biomarker survival analysis was conducted, in which random selection was used to select five biomarkers from each group. As indicated in Fig. 3, there is an evident difference in both publication number and publication window between the two groups. In specific, successful biomarkers (Fig. 3A–E) appear to have more published papers per year, over a longer period of time than stalled ones (Fig. 3A–E vs F–J).

As seen in Fig. 3H, the first published paper regarding Gasdermin was in 2015. To account for the fact that some biomarkers are relatively new (recently published), and therefore did not have time to accumulate interest; the time in (years) between the biomarker’s first clinical study and adoption/recommendation date was used to re-calculate the percentage of successful biomarkers. Specifically, the median years of adoption between the first publication and the date of recommendation/adoption for all successful biomarkers was 6 years. Thus, all biomarkers with a first clinical publication from 2017 onwards (2017 = 2023–6) were marked and excluded from the % successful biomarker calculation. Upon removal of these biomarkers from the total number of identified biomarkers, the percentage survival increased from 0.94 to 1.42% (< 2%, successful BMs:23 vs stalled BMs:2414).

**Fig. 4:**
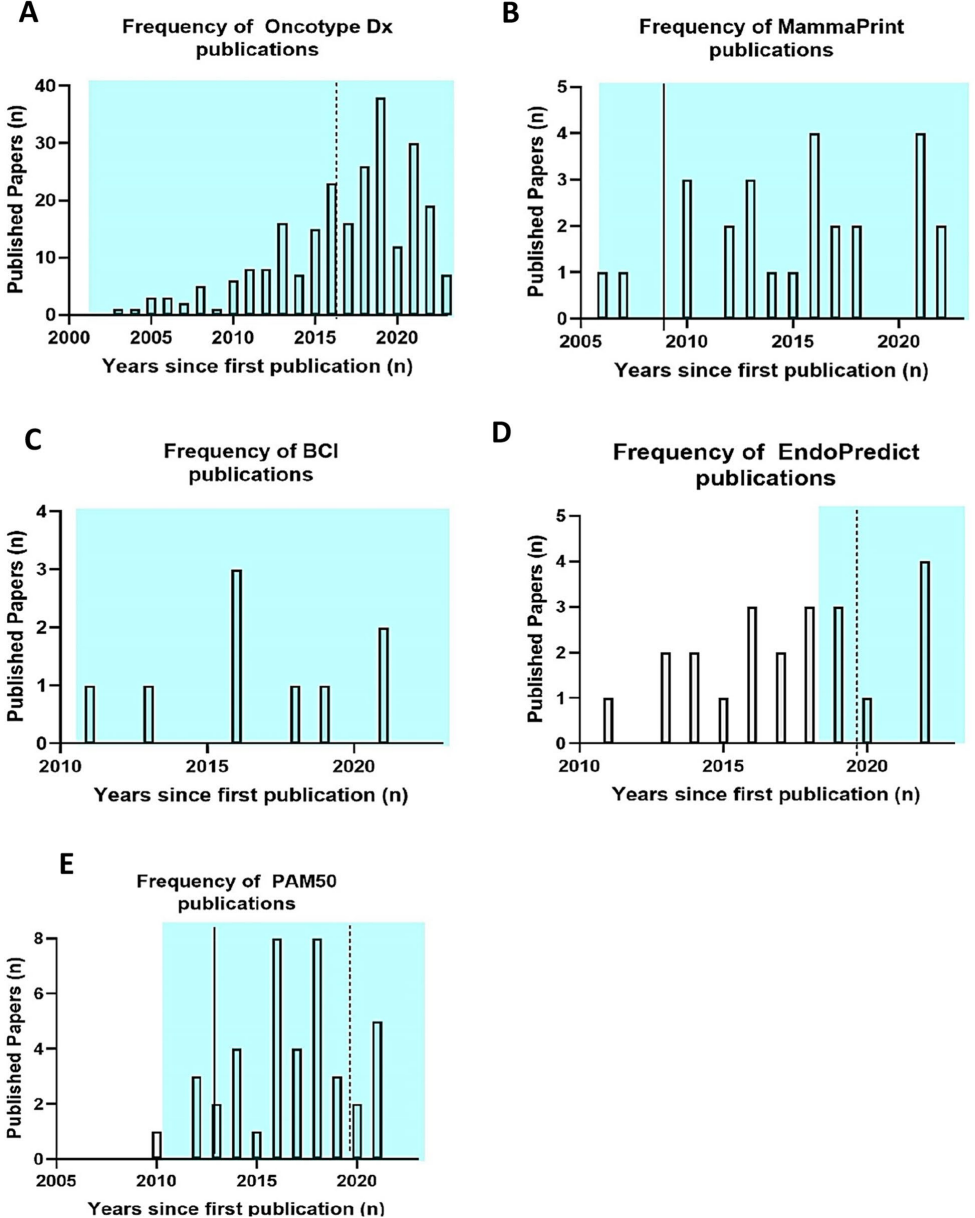
Commercialisation, authority approval and biomarker success. Bar charts indicating the number of published papers over the years for **A** Oncotype Dx, **B** MammaPrint, **C** BCI, **D** Endopredict and **E** PAM50. These biomarkers were the only biomarkers identified to be commercialised. All of the stated biomarkers are recommended by guidelines/regulatory authorities. BCI is not currently approved by FDA/NICE, but is recommended by NCCN, ASCO and EGTM. The period of biomarker test commercialisation is indicated by the light blue window, the NICE approval date is denoted by the broken line while the FDA date of approval is illustrated by a continuous line. PAM50 (also referred as Prosigna): Prediction Analysis of Microarray 50, BCI, Breast Cancer Index

The influence of biomarker test commercialisation and regulatory authority approval on publication frequency was also examined. Biomarkers presented in Fig. 4 were selected as they were the only commercialised biomarkers. As seen in Fig. 4, test commercialisation, as well as FDA & NICE approval, do not evidently result in altered publication frequency.

cBioPortal was utilised to assess prognosis-related outcomes in all successful and stalled biomarkers with more than 12 publications (Additional file: Fig. S1). As indicated in Additional file: Fig. S2, *P*-values for disease-free survival, disease-specific survival, progression-free survival, relapse-free survival and overall survival appear to have similar distributions between stalled and successful biomarkers. *P*-values extracted assess the extent of difference in outcomes between the mutated and wildtype version of the biomarker, in each cohort. Binary logistic regression illustrated no significant association between reported *P* values for each prognostic outcome and biomarker clinical implementation status (see Additional file: Fig. S2 and Table S4).

## Discussion

The gap between biomarker discovery and clinical translation is large. The aim of this novel study is to quantitively characterise this gap using recurrence-related breast cancer biomarkers. Key outcomes of this study include (i) a high rate of discrepancy between biomarker discovery and clinical translation as only 0.94% of identified biomarkers are currently recommended for clinical use by guidelines/regulatory authorities and (ii) a significantly higher frequency of publications, published over a larger time-frame and a higher impact factor for successful biomarker publications. Other than assessing the relation of these factors with biomarker success, this study reinforces the need to identify additional features that would enable researchers to assess a biomarker’s potential for clinical implementation.

The recurrence breast cancer biomarker translation rate was critically low, 0.94%. Consistently low clinical implementation rates in the literature support our findings and emphasise the lack of efficiency within the biomarker pipeline [19, 20]. Potential barriers of biomarker implementation are well documented in the literature. Numerous impediments inhibiting the discovery and development of biomarkers can be attributed to incomplete, inappropriate, or inadequately reported scientific findings. These barriers encompass a complex array of factors, including insufficient sample size, inappropriate statistical methodologies, absence of clinical relevance, inadequate characterisation of study cohorts, limited analytical validation, biological variability, and potential biases stemming from sponsor influence, among others [21, 22]. Although numerous studies highlight the translation barriers and the low number of clinically used biomarkers, only a few of them quantitively assess this [23, 24], emphasising the importance of the current study.

Assuming that successful biomarkers would have a larger evidence base due to greater scientific interest, the relationship of biomarker success and publication frequency was explored. In support, 91.67% of the successful biomarkers were in the > 20 publication category (Fig. 2C). This is in stark contrast to the stalled biomarker group in which only 0.29% were in the > 20 publication category. Moreover, 77.34% of stalled biomarkers had a single publication. This reinforces the concept that there is a high rate of biomarker discovery taking place in academia, but very little progression of these biomarkers towards clinical utilisation, further supporting the literature as well as the study hypothesis [25].

As expected, successful biomarkers were significantly associated with higher publication number, using binary logistic regression (publication frequency: *p* = 0.002 (95% CI: (0.904–0.978)), with a more consistent increase in publications over time, as indicated in Fig. 3. The low frequency of published literature in the stalled biomarker group could be due to a lack of biomarker efficacy in predicting recurrence or lack of standardised biomarker assay employment. However, drawing conclusions on this seems unfounded as we have not analysed the strength of the relationship between biomarkers and recurrence, while the cBioPortal analysis performed seems to suggest no differences between the groups (Additional file: Fig. S2).

Low publication frequency in certain cases might be because some biomarkers are newer than others; therefore, the date of publication should also be considered. Considering the influence of this factor, publication frequency within a 5- and 10-year window was evaluated. Within the 5-year window, at least two papers were published in the successful biomarker group, while most of the stalled biomarkers had a single publication. This supports our finding that Fascin and Synuclein might be promising biomarkers, as they have a larger number of biomarker publications (Fig. 3G and J). Almost all of the successful biomarkers had > 6 publications (Fig. 3), in contrast the stalled group which at most cases (SPG130, (TA)9 repeats and Gasdermin) published only a single paper within 10 years. Nevertheless, the 10-year window comparison was not possible for all of the biomarkers, since some of them were published less than 10 years ago. For example, Gasdermin (Fig. 3H) will naturally have a lower number of publications as this biomarker has not yet had time to stimulate interest in the biomedical community. To account for this confounder, all biomarkers with a first clinical publication from 2017 onwards were excluded from the % successful biomarker calculation, as described in the result section. This resulted in a slight improvement in the % biomarker translation rate, from 0.94 to 1.42%, which is still extremely low. Moreover, 3 (0.43%) of the biomarkers discovered from ≥ 2017 had five publications, supporting that these biomarkers could be promising, as they have increased interest.

The FDA biomarker Qualification Framework stresses the importance of a strong relationship between biomarker and outcome, sample size and reproducibility of results [26]. Naturally, a thorough assessment of these analytical validity parameters requires numerous studies. This may explain the positive relationship between publication frequency and success [27]. This finding can in part be because biomarkers featured in guidelines would raise awareness and interest in a biomarker. A solid evidence base would also need to be initially present for the biomarker to be approved. This pattern is supported by Fig. 3 where both Oncotype Dx and Endopredict accumulated numerous publication evidence prior to approval. However, as indicated in Fig. 4B some biomarkers may reach success after a relatively low number of publications. This demonstrates that biomarker success cannot always be predicted by publication frequency and distribution in isolation. Assessing the frequency of biomarker publication and correlating this to biomarker success was an initial attempt to detect factors associated with successful biomarker implementation. As supported by cBioPortal data, there was no significant difference between the clinical performance of stalled and successful biomarkers (Additional file: Fig. S2). This supports the finding that the performance of a biomarker reported in literature does not clearly indicate how promising a biomarker will be for clinical use. This reinforces the role of the Biomarker Toolkit, a novel tool that provides quantitative biomarker evaluation by considering the accuracy of reporting in four pillars including rationale, analytical validity, clinical validity, and clinical utility [7]. The output generated by this tool has the potential to predict biomarker success and direct future investigations towards evaluating supplementary attributes, such as the nature of the study (e.g., preclinical/in vitro studies, clinical trials, high-throughput prospective or retrospective biomarker discovery studies). This expanded analysis could enhance the predictive capacity for biomarker success and consequently direct research interest.

Biomarker commercialisation is a vital step in the biomarker development; hence we also investigated the relation of commercialisation with publication frequency. Interestingly, Mammaprint showed a rapid rise of published literature upon FDA approval. Nevertheless, this pattern might be due to the fact that MammaPrint was commercialised very early. Commercialisation fortifies test standardisation and provides a promising factor that could be linked with biomarker clinical implementation. Hence, it would be logical to assume that biomarker commercialisation would attract additional interest [28]. However, current data indicated that publication density does not appear to change dramatically upon guideline recommendation or commercialisation. This might be due to a lack of accurate reporting or insufficient published data prior to test commercialisation to allow assessment of publication frequency. Interestingly, IHC4, demonstrated a similar pattern to MammaPrint in terms of publications (Additional File: Fig. S1), however, although assessed by guidelines, it is not currently recommended mainly due to the limited reproducibility/analytical validation of the immunohistochemical technique used to assess this biomarker (NICE guidelines). This reinforces the idea that methodological and analytical validation aspects should be addressed at an early state to enhance biomarker clinical translation. Overall, commercialisation and authority approval do not appear to have an evident association with publication frequency.

Due to the large numbers of stalled biomarkers with minimal publications, the Excel random function selected stalled biomarkers with lower publication frequencies (Fig. 3). However, there is a minor subset of biomarkers which have over 20 publications (see Additional File: Table S2). Although these biomarkers are not currently clinically used, the high level of research papers suggests that scientists, clinicians, and industry should study the evidence of these biomarkers closely and assess the reasons which have prevented them from being clinically adopted. As emphasised by many authors potential biomarkers might lack a robust analytical methodology or evaluation studies, including cost-effectiveness and feasibility studies. Therefore, this study reinforces the fact that biomarker progress should be studied in detail to identify areas of improvement that could promote their adoption in the clinic, emphasising the importance of the Biomarker Toolkit [7].

In support of the hypothesis, the journal impact factor was significantly higher for successful biomarkers in comparison to stalled (*p* < 0.0001). The highest impact factor for each biomarker was used to assess the biomarkers at peak levels of research quality and interest, where publications have been subjected to rigorous peer review. The highest impact factor publication noted was 51.77, a biomarker published in the Annals of Oncology in 2021 (Ki-67) [18]. This publication fulfilled many of the criteria suggested by the FDA for biomarker translation success including large sample size, defined cohort, clinical intent, and powered statistics indicating that, in this case, high impact factor reflects high-quality research publication [29]. However, whilst the journal impact factor is useful as an overview of publication quality and academic success, it should be interpreted with caution as the score is subject to artificial inflation by practice of self-citation [30, 31]. Therefore, whilst it is useful to get a broad overview of interest a biomarker is attracting, it is not necessarily the optimal measure to assess research quality [32].

Whilst this study identifies candidate biomarkers and qualities that are likely associated with their successful clinical utility, it does not discriminate between the strength of evidence/ association with recurrence of each biomarker. Future research may also utilise the Biomarker Toolkit to score biomarkers. This will eventually assist in prioritising research into those biomarkers with increased chances of clinical utilisation. These data could be integrated within a centralised database which might prioritise future research, keeping scientists and clinicians up to date on biomarker discovery [33]. Comprehending the factors influencing the clinical utility of biomarkers is poised to steer research focus, mitigate redundant exploration and its associated costs, enhance the rate of biomarker translation, and, most importantly, contribute significantly to improving patient outcomes [33].

Nevertheless, this novel study also suffers from some limitations. Specifically, abstracts alone were screened and articles were rejected if insufficient information was present. This may be inappropriate since many journals have word-limited abstract content and therefore crucial information necessary to fulfil the inclusion criteria may have been presented in the full-text only. Due to the extremely large number of publications, full-text screening would be unrealistic considering the time scale of the study. Furthermore, this study focuses on recurrence-related breast cancer biomarkers; future work should aim to assess the transitional rate of additional biomarker types for other cancer types. Focus was directed on recurrence rather than overall patient prognosis, to maintain a realistic number of Ovid results.

This study defined successful biomarkers, as those approved/recommended by regulatory bodies and guidelines. While exploring previously mentioned guidelines (see Table 1), we identified that different sets of biomarkers were recommended in some cases, and in many instances, it was difficult to trace up-to-date published recommendations. In certain cases, there was no clear repository of recommended biomarkers associated with recurrence. This emphasises the need for better reporting and co-ordination between the different bodies to develop a user-friendly repository with biomarker recommendations, for different cancer and biomarker types.

## Conclusions

In summary, this study is the first attempt at (i) assessing the percentage of clinically useful breast cancer biomarkers directly/indirectly associated with recurrence and (ii) in evaluating the relationship between impact factor and publication frequency with biomarker clinical implementation. Future work will aim to examine in detail biomarker publications to define literature-reported characteristics associated with a clinically useful biomarker. This will allow biomarker assessment and enable researchers to (i) critically evaluate biomarker research and (ii) identify biomarkers that are more likely to be clinically useful. Thus, research interest and funding could then be directed in a targeted manner, minimising the translational gap and ultimately improving patient care.

### Supplementary Information

Supplementary Material 1: Supplementary Fig. 1. Biomarker Survival Analysis: Bar charts indicating the number of published papers over the years for IHC4. Supplementary Fig. 2. Evaluating differences between stalled and successful biomarker prognostic outcomes using cBioPortal: Survival outcomes of all successful and stalled biomarkers with more than 12 publications were assessed. *P*-values comparing the survival outcome between the mutant and wildtype version of the biomarker from 16 breast cancer studies (6805 breast samples/6391 patients) were extracted. Supplementary Table 1. Search terms used to assess biomarker success rate. Supplementary Table 2. Stalled BMs with greater than 20 publications. Supplementary Table 3. cBioPortal Studies included in the analysis. Supplementary Table 4. Binary logistic Regression Results assessing the relationship between biomarker success and prognostic outcomes.

## Data Availability

All data presented in the study can be found in the main and Additional file document. Additional raw Data are available upon request.

## Abbreviations

BCI: Breast Cancer Index
BRCA1/2: BReast CAncer gene 1/2
CA 27-29: Cancer antigen 27–29
CA15-3: Cancer antigen 15–3
CEA: Carcinoembryonic antigen
CTC: Circulating tumour cells
EGTM: European Group on Tumour Markers
ER: Estrogen receptors
FDA: Food and Drug Administration
HER2: Human epidermal growth factor receptor 2
NCCN: National Comprehensive Cancer Network
OncoDx: OncotypeDx
P53: Tumour protein p53
PAI-1: Plasminogen activator inhibitor type-1
PAM50 (also referred as Prosigna): Prediction Analysis of Microarray 50
PDL1: Programmed death-ligand 1
PIK3CA: Phosphatidylinositol-4,5-bisphosphate 3-kinase catalytic subunit alpha
PR: Progesterone receptors
SPG130: Soluble glycoprotein
TILs: Tumour-infiltrating lymphocytes
TNBC: Triple negative breast cancer
UPA: Urokinase plasminogen activator
