# Supplementary material for: An original study assessing biomarker success rate in breast cancer recurrence biomarker research
Source: BMC Med. 2024 Jul 29;22:307. doi: 10.1186/s12916-024-03460-6 (PMC11288100; doi:10.1186/s12916-024-03460-6)
Supplement: Supplementary file 1 — Supplementary Material 1: Supplementary Fig. 1. Biomarker Survival Analysis: Bar charts indicating the number of published papers over the years for IHC4. Supplementary Fig. 2. Evaluating differences between stalled and successful biomarker prognostic outcomes using cBioPortal: Survival outcomes of all successful and stalled biomarkers with more than 12 publications were assessed. P-values comparing the survival outcome between the mutant and wildtype version of the biomarker from 16 breast cancer studies (6805 breast samples/6391 patients) were extracted. Supplementary Table 1. Search terms used to assess biomarker success rate. Supplementary Table 2. Stalled BMs with greater than 20 publications. Supplementary Table 3. cBioPortal Studies included in the analysis. Supplementary Table 4. Binary logistic Regression Results assessing the relationship between biomarker success and prognostic outcomes. [file 12916_2024_3460_MOESM1_ESM.docx]

**SUPPLEMENTARY MATERIAL**

| **Supplementary Table 1**: Search terms used to assess biomarker success rate | | | | | |
| --- | --- | --- | --- | --- | --- |
|  | **Embase** |  |  | **Medline** |  |
| 1 | exp breast tumor/ | 660,988 | 1 | exp breast neoplasms/ | 348,200 |
| 2 | exp breast cancer/ | 580,489 | 2 | Breast neoplasm*.mp. | 349,683 |
| 3 | exp breast carcinoma/ | 101,519 | 3 | Breast tumo?r*.mp. | 28,186 |
| 4 | Breast neoplasm*.mp. | 19,792 | 4 | Breast carcinoma*.mp. | 33,612 |
| 5 | Breast tumo?r*.mp. | 121,793 | 5 | Breast cancer*.mp. | 353,039 |
| 6 | Breast carcinoma*.mp. | 96,958 | 6 | Mammary neoplasm*.mp. | 23,632 |
| 7 | Breast cancer*.mp. | 650,099 | 7 | Mammary tumo?r*.mp. | 16,900 |
| 8 | Mammary neoplasm*.mp. | 1,398 | 8 | Mammary cancer*.mp. | 3,739 |
| 9 | Mammary tumo?r*.mp. | 19,775 | 9 | Breast carcinogenesis.mp. | 1,801 |
| 10 | Mammary cancer*.mp | 5,568 | 10 | exp BMs, tumor/ | 312,489 |
| 11 | Breast carcinogenesis.mp. or exp breast carcinogenesis | 13,468 | 11 | BM*.mp. or exp BMs | 1,144,719 |
| 12 | exp tumor marker/ | 392,990 | 12 | Cancer BM*.mp. | 7,202 |
| 13 | exp biological marker/ or BM*.mp. | 525,268 | 13 | exp neoplasm recurrence, Local/ or cancer recurrence.mp. | 152,605 |
| 14 | Cancer BM*.mp. | 6,751 | 14 | exp recurrence/ or recurrence.mp | 594,634 |
| 15 | Cancer recurrence.mp. or exp cancer recurrence/] | 191,798 | 15 | relapse.mp. | 151,262 |
| 16 | Recurrence.mp. or exp recurrent disease/ | 729,386 | 16 | cancer relapse.mp. | 1,314 |
| 17 | relapse/ or relapse.mp. | 341,457 | 17 | 1 or 2 or 3 or 4 or 5 or 6 or 7 or 8 or 9 | 483,369 |
| 18 | cancer relapse.mp. or exp cancer relapse/ | 289,297 | 18 | 10 or 11 or 12 | 1,144,719 |
| 19 | carci* relapse.mp. or exp carci* relapse/ | 90 | 19 | 13 or 14 or 15 or 16 | 689,660 |
| 20 | 1 or 2 or 3 or 4 or 5 or 6 or 7 or 8 or 9 or 10 or 11 | 745,698 | 20 | 17 and 18 and 19 | 8,830 |
| 21 | 15 or 16 or 17 or 18 | 909,698 |  |  |  |
| 22 | 12 or 13 or 14 | 824,986 |  |  |  |
| 23 | 20 and 21 and 22 | 10,365 |  |  |  |


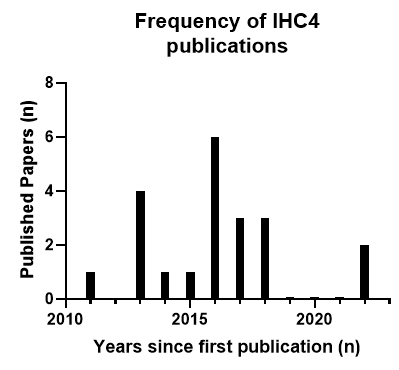


**Supplementary Fig. 1**: **Biomarker Survival Analysis**: Bar charts indicating the number of published papers over the years for IHC4

| **Supplementary Table 2: Stalled BMs with greater than 20 publications.** | |
| --- | --- |
| **Biomarker Name** | **Total Published Literature** |
| IHC4 | 21 |
| CD44 | 21 |
| Cyclin E | 21 |
| PTEN | 21 |
| E-cadherin | 23 |
| ALDH1 | 24 |
| TOP2A | 29 |
| VEGF | 33 |
| BCL2 | 39 |
| AR | 47 |
| EGFR | 49 |
| IHC4: Immunohistochemistry 4, PTEN: Phosphatase and Tensin Homolog, ALDH1: Aldehyde Dehydrogenase 1, TOP2A: DNA Topoisomerase II Alpha , VEFG: Vascular Endothelial Growth Factor, BCL2: B-cell lymphoma 2, AR: Androgen receptor, EGFR: Epidermal Growth Factor Receptor | |

**Supplementary Fig. 2**: **Evaluating differences between stalled and successful biomarker prognostic outcomes using cBioPortal:** Survival outcomes of all successful and stalled biomarkers with more than 12 publications were assessed. P-values comparing the survival outcome between the mutant and wildtype version of the biomarker from 16 breast cancer studies (6805 breast samples/6391 patients) were extracted.

.

| **Supplementary Table 3**: cBioPortal Studies included in the analysis |
| --- |
|  |
| This combined study contains samples from 16 studies |
| [Breast Cancer (MSK, Cancer Cell 2018)](https://www.cbioportal.org/study?id=breast_msk_2018) |
| [Breast Cancer (MSK, Nature Cancer 2020)](https://www.cbioportal.org/study?id=breast_alpelisib_2020) |
| [Breast Fibroepithelial Tumors (Duke-NUS, Nat Genet 2015)](https://www.cbioportal.org/study?id=bfn_duke_nus_2015) |
| [Breast Cancer (METABRIC, Nature 2012 & Nat Commun 2016)](https://www.cbioportal.org/study?id=brca_metabric) |
| [Breast Cancer (MSKCC, NPJ Breast Cancer 2019)](https://www.cbioportal.org/study?id=brca_mskcc_2019) |
| [Breast Cancer (SMC 2018)](https://www.cbioportal.org/study?id=brca_smc_2018) |
| [Breast Invasive Carcinoma (British Columbia, Nature 2012)](https://www.cbioportal.org/study?id=brca_bccrc) |
| [Breast Invasive Carcinoma (Broad, Nature 2012)](https://www.cbioportal.org/study?id=brca_broad) |
| [Breast Cancer Xenografts (British Columbia, Nature 2015)](https://www.cbioportal.org/study?id=brca_bccrc_xenograft_2014) |
| [Breast Invasive Carcinoma (Sanger, Nature 2012)](https://www.cbioportal.org/study?id=brca_sanger) |
| [Juvenile Papillomatosis and Breast Cancer (MSK, 2020)](https://www.cbioportal.org/study?id=brca_jup_msk_2020) |
| [Metastatic Breast Cancer (INSERM, PLoS Med 2016)](https://www.cbioportal.org/study?id=brca_igr_2015) |
| [The Metastatic Breast Cancer Project (Provisional, February 2020)](https://www.cbioportal.org/study?id=brca_mbcproject_wagle_2017) |
| [Breast Invasive Carcinoma (TCGA, PanCancer Atlas)](https://www.cbioportal.org/study?id=brca_tcga_pan_can_atlas_2018) |
| [Metaplastic Breast Cancer (MSK, 2021)](https://www.cbioportal.org/study?id=mbc_msk_2021) |
| [Adenoid Cystic Carcinoma of the Breast (MSKCC, J Pathol. 2015)](https://www.cbioportal.org/study?id=acbc_mskcc_2015) |

| **Supplementary Table 4**: Binary logistic Regression Results assessing the relationship between biomarker success and prognostic outcomes | | | | |
| --- | --- | --- | --- | --- |
| **Variables in the equation** | **Sig.** | **Exp(B)** | **Lower** | **Upper** |
| Disease free-Survival | 0.173 | 0.033 | 0.000 | 4.452 |
| Disease Specific-Survival | 0.094 | 31.990 | 0.557 | 1837.829 |
| Overall Survival | 0.851 | 0.664 | 0.009 | 47.068 |
| Progression Free Survival | 0.400 | 3.288 | 0.205 | 52.718 |
| Relapse Free Survival | 0.482 | 0.468 |  |  |
